# Noninferior Outcome After Breast-Conserving Treatment Compared to Mastectomy in Breast Cancer Patients With Four or More Positive Lymph Nodes

**DOI:** 10.3389/fonc.2019.00143

**Published:** 2019-03-12

**Authors:** Jun Wang, Jia-Peng Deng, Jia-Yuan Sun, Yong Dong, Wen-Wen Zhang, Zhen-Yu He, San-Gang Wu

**Affiliations:** ^1^Department of Radiation Oncology, Cancer Hospital, The First Affiliated Hospital of Xiamen University, Teaching Hospital of Fujian Medical University, Xiamen, China; ^2^State Key Laboratory of Oncology in South China, Department of Radiation Oncology, Sun Yat-sen University Cancer Center, Collaborative Innovation Center of Cancer Medicine, Guangzhou, China; ^3^Department of Oncology, Dongguan Third People's Hospital, Affiliated Dongguan Shilong People's Hospital of Southern Medical University, Dongguan, China

**Keywords:** invasive breast cancer, breast conserving treatment, mastectomy, radiotherapy, node positive

## Abstract

**Introduction:** We conducted a non-inferiority analysis using real-world data to compare the survival outcomes of stage T1-2N2-3 (tumor size ≤5 cm and four or more node metastases) breast cancer after breast-conserving surgery (BCS) and mastectomy (MAST).

**Methods:** The study included patients with stage T1-2N2-3 invasive breast carcinoma from the Surveillance, Epidemiology, and End Results program, who underwent BCS or MAST between 2004 and 2012, along with both radiotherapy and chemotherapy. The statistical analyses used included the chi-squared test, multivariate Cox proportional hazards models, and propensity score matching (PSM).

**Results:** The study population comprised 13,263 patients, including 4,787 (36.1%) and 8,476 (63.9%) patients who were treated with BCS and MAST, respectively. Patients with younger age and advanced stage were more likely to have received MAST. The probability of receiving MAST increased over the years, while the probability of BCS decreased (*p* < 0.001). The 5-year breast cancer-specific survival (BCSS) was 86.1% in the BCS cohort compared to 83.1% in the MAST cohort (*p* < 0.001). Surgical procedure was an independent prognostic factor for BCSS. Patients who received MAST had worse BCSS than those treated with BCS (hazard ratio = 1.179, 95% confidence interval = 1.087–1.278, *p* < 0.001). These results remained significant after stratification by age, tumor grade, T stage, N stage as well as marital status. Similar results were obtained after PSM.

**Conclusions:** BCS resulted in noninferior outcome than MAST in patients with T1-2/N2-3 invasive breast carcinoma. BCS may therefore be an optimal treatment option when both treatment options are feasible and appropriate.

## Background

Breast cancer is a major health concern in women, as approximately 2 million new breast cancer patients are diagnosed worldwide annually (1). As a result of all the advances in diagnostic and screening techniques, most cases of breast cancer are diagnosed in the early stages. Despite this, 10% of patients are diagnosed with high-risk breast cancer (four or more node metastases) in developed countries (2, 3), and this percentage is as high as 15–20% in developing countries (4, 5). Lymph node status is the main prognostic factor that affects the outcome of breast cancer, and patients with four or more node metastases (N2-3) have significantly worse survival than those who have no node metastases (N0) or one to three node metastases (N1) (6).

With regard to N0 and N1 breast cancer, studies from prospective and large-scale retrospective studies have confirmed that breast-conserving surgery (BCS) has similar and even superior outcomes to mastectomy (MAST) (9–15). In contrast, the current standard treatment strategies for patients with stage N2-3 breast cancer are local surgery plus chemotherapy and postoperative adjuvant radiotherapy (RT), endocrine therapy for hormone receptor (HoR)-positive patients, and targeted therapy for human epidermal growth factor receptor-2 (**HER2**)-positive tumors (7, 8). The usefulness of BCS in patients with N2-3 breast cancer remains unclear, especially for those in the N3 class, which is often considered as a contraindication for BCS (16). This is probably because in patients with N2-3 disease, the risk of local recurrence and distant metastasis is significantly higher than that in patients with N0 and N1 disease (6). No prospective studies or randomized controlled trials so far have compared the survival outcome of surgical treatment of N2-3 breast cancer. Further, although several retrospective studies have shown that BCS has superior outcomes to MAST in N2-3 breast cancer, the adjuvant treatment provided, including chemotherapy and RT, was found to be insufficient in most cases, which might have affected the evaluation of the results (2, 14, 17, 18). There is clearly a need to analyze these findings in a population of N2-3 breast cancer patients who underwent BCS and also received appropriate adjuvant treatment.

The present study sought to contribute to the knowledge about the efficiency of BCS for N2-3 breast cancer by conducting a non-inferiority analysis using real-world data to assess the long-term effects of BCS in patients with T1-2N2-3 (tumor size ≤5 cm and four or more node metastases) breast cancer who underwent BCT or MAST, along with chemotherapy and RT.

## Materials and Methods

### Study Population

In this study, patients with pathologically diagnosed T1-2N2-3M0 invasive breast cancer treated with BCS+postoperative beam RT+chemotherapy or MAST+postoperative beam RT+chemotherapy between 2004 and 2012 were included from the population-based Surveillance, Epidemiology, and End Results (SEER) program (19). The SEER program is headed by the National Cancer Institute, which maintains a database for de-identified cancer incidence, demographic and clinicopathological variables, first course of treatment as well as survival, through 18 cancer registries across the United States. Patients with insufficient data on race/ethnicity, tumor grade, HoR status, and marital status were excluded. As the SEER registries contain de-identified information about patients, this study was exempt from the approval process of the Institutional Review Board.

### Procedures

Data on demographic, clinicopathological, and treatment-related variables as well as vital status were obtained from the SEER program. The variables included age (<50 years, ≥50 years), race/ethnicity (non-Hispanic white, Non-Hispanic clack, Hispanic, other), tumor grade (well differentiated, moderately differentiated, poorly differentiated/undifferentiated), T stage (T1, T2), N status (N2, N3), HoR status (estrogen receptor [ER]+ and progesterone receptor [PR]+, ER+, or PR+, ER- and PR-), marital status (unmarried, married), and surgical procedure (BCS, MAST). Data on HER2 status were not routinely registered before 2010, so HER2 status was not included in this study. The primary outcome of this study was breast cancer-specific survival (BCSS).

### Statistical Analysis

The difference in categorical variables between the two treatment arms (MAST and BCS) were compared using the chi-squared test. A propensity score matching (PSM) method was used to reduce the potential confounding factors and selection bias between each group (20, 21). A 1:1 PSM cohort was created using the above 7 including variables, which could potentially be confounding factors. Long-term BCSS was assessed and compared by the Kaplan–Meier method and log-rank test. The variables that could influence BCSS were analyzed using the multivariate Cox proportional hazard model, and the risk for BCSS was assessed by calculating the hazard ratio (HR) and the corresponding 95% confidence interval (CI). Statistical analyses were conducted using IBM SPSS version 22.0 (IBM Corp., Armonk, NY), and a *p* < 0.05 was considered to indicate statistical significance.

## Results

### Demographic and Clinical Data

We included 13,263 patients who met the inclusion criteria (median age = 54 years). The patient characteristics are listed in Table 1. Two-thirds of the patients were aged ≥50 years (65.2%, *n* = 8,652), and the group comprised 8,936 (67.4%) non-Hispanic white patients, 9,075 (68.4%) patients with T2 stage cancer, 9,202 (69.4%) patients with N2 stage cancer, 8,161 (61.5%) ER+ and PR+ patients, and 8,316 (62.7%) married patients.

**Table 1:** Patient characteristics according to treatment arm.

**Variables**	***n***	**BCS (%)**	**MAST (%)**	***p***
**AGE (YEARS)**				
< 50	4,611	1,490 (31.1)	3,121 (36.8)	< 0.001
≥50	8,652	3,297 (68.9)	5,355 (63.2)	–
**RACE/ETHNICITY**				
Non-Hispanic white	8,936	3,228 (67.4)	5,708 (67.3)	0.002
Non-Hispanic black	1,641	645 (13.5)	996 (11.8)	–
Hispanic (all races)	1,613	568 (11.9)	1,045 (12.3)	–
Other	1,073	346 (7.2)	727 (8.6)	–
**GRADE**				
Well differentiated	1,033	382 (8.0)	651 (7.7)	0.813
Moderately differentiated	5,291	1,901 (39.7)	3,390 (40.0)	–
Poorly differentiated/undifferentiated	6,939	2,504 (52.3)	4,435 (52.3)	–
**TUMOR STAGE**				
T1	4,188	1,981 (41.4)	2,207 (26.0)	< 0.001
T2	9,075	2,806 (58.6)	6,269 (74.0)	–
**NODAL STATUS**				
N2	9,202	3,538 (73.9)	5,664 (66.8)	< 0.001
N3	4,061	1,249 (26.1)	2,812 (33.2)	–
**HORMONE RECEPTOR STATUS**				
ER+ and PR+	8,161	2,955 (61.7)	5,206 (61.4)	0.053
ER+ or PR+	1,927	652 (13.6)	1,275 (15.0)	–
ER- and PR-	3,175	1,180 (24.7)	1,995 (23.5)	–
**MARITAL STATUS**				
Unmarried	4,947	1,833 (38.3)	3,114 (36.7)	0.076
Married	8,316	2,954 (61.7)	5,362 (63.3)	–

*BCS, breast conserving surgery; ER, estrogen receptor; MAST, mastectomy; N, node; PR, progesterone receptor; T, tumor*.

### Treatment Data

**Figure 1:**
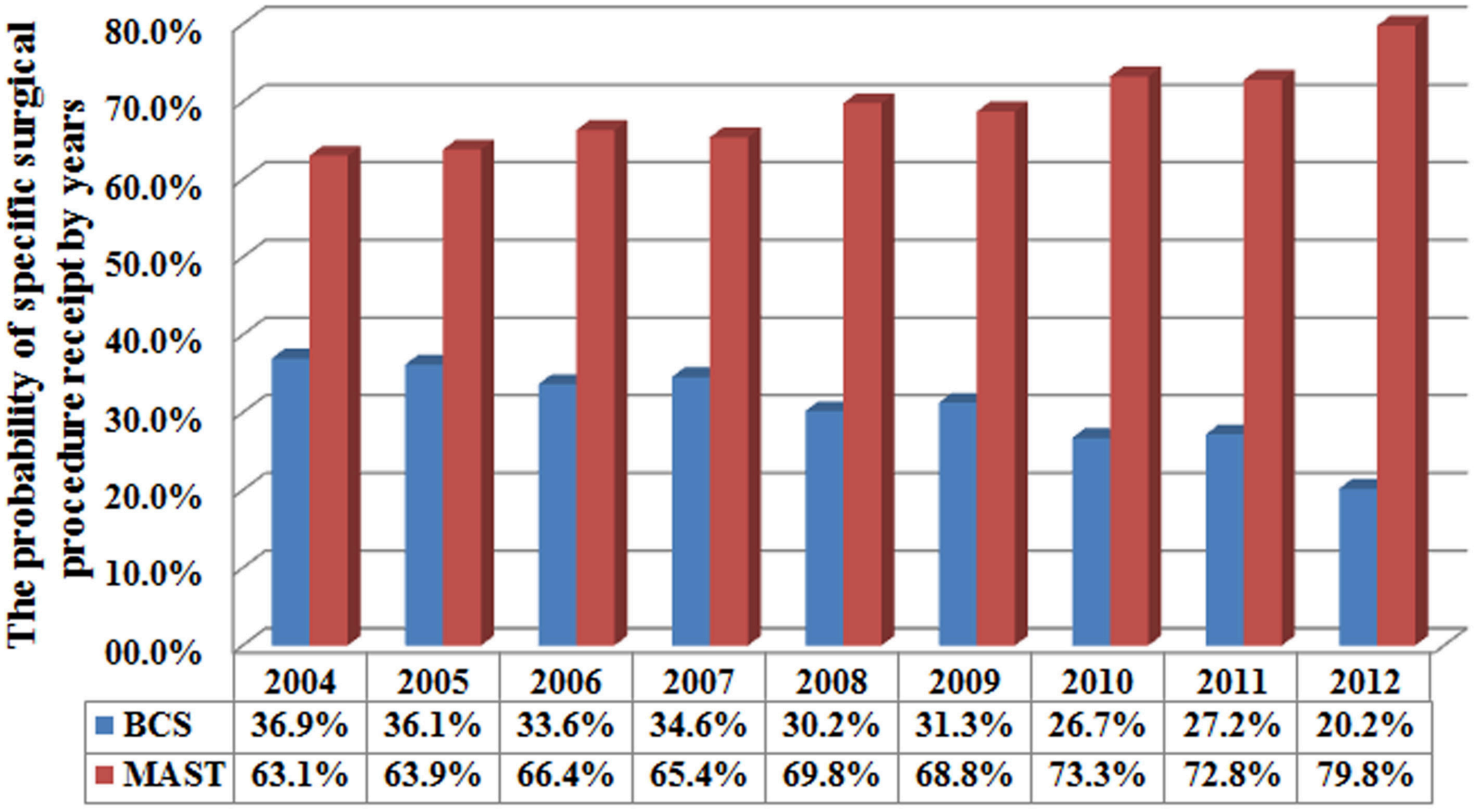
Utilization of breast-conserving surgery and mastectomy during the studied period.

**Figure 2:**
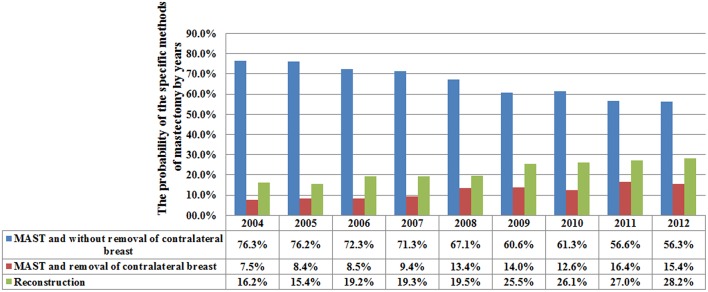
Utilization of specific mastectomy procedures during the studied period.

A total of 4,787 (36.1%) and 8,476 (63.9%) patients were treated with BCS and MAST, respectively. Patients with younger age, T2, and N3 stage disease were more likely to have undergone MAST. Figure 1 lists the temporal trends in surgical procedure from 2004 to 2012: notably, the probability of undergoing MAST increased over the years, while the probability of BCS decreased over the years (*p* < 0.001). Data about specific treatment methods for 8,036 patients who underwent MAST were available: 5,322 (66.2%), 950 (11.8%), and 1,764 (22.0%) patients underwent MAST without removal of the contralateral breast, MAST along with removal of the contralateral breast, and MAST along with tissue and/or implant reconstruction, respectively. Figure 2 lists the temporal trends in the specific surgical procedures used for MAST from 2004 to 2012: notably, the probability of MAST without removal of the contralateral breast significantly decreased over the years, while the probability of MAST along with removal of the contralateral breast and reconstruction significantly increased over the years (*p* < 0.001).

### Treatment Outcomes

**Figure 3:**
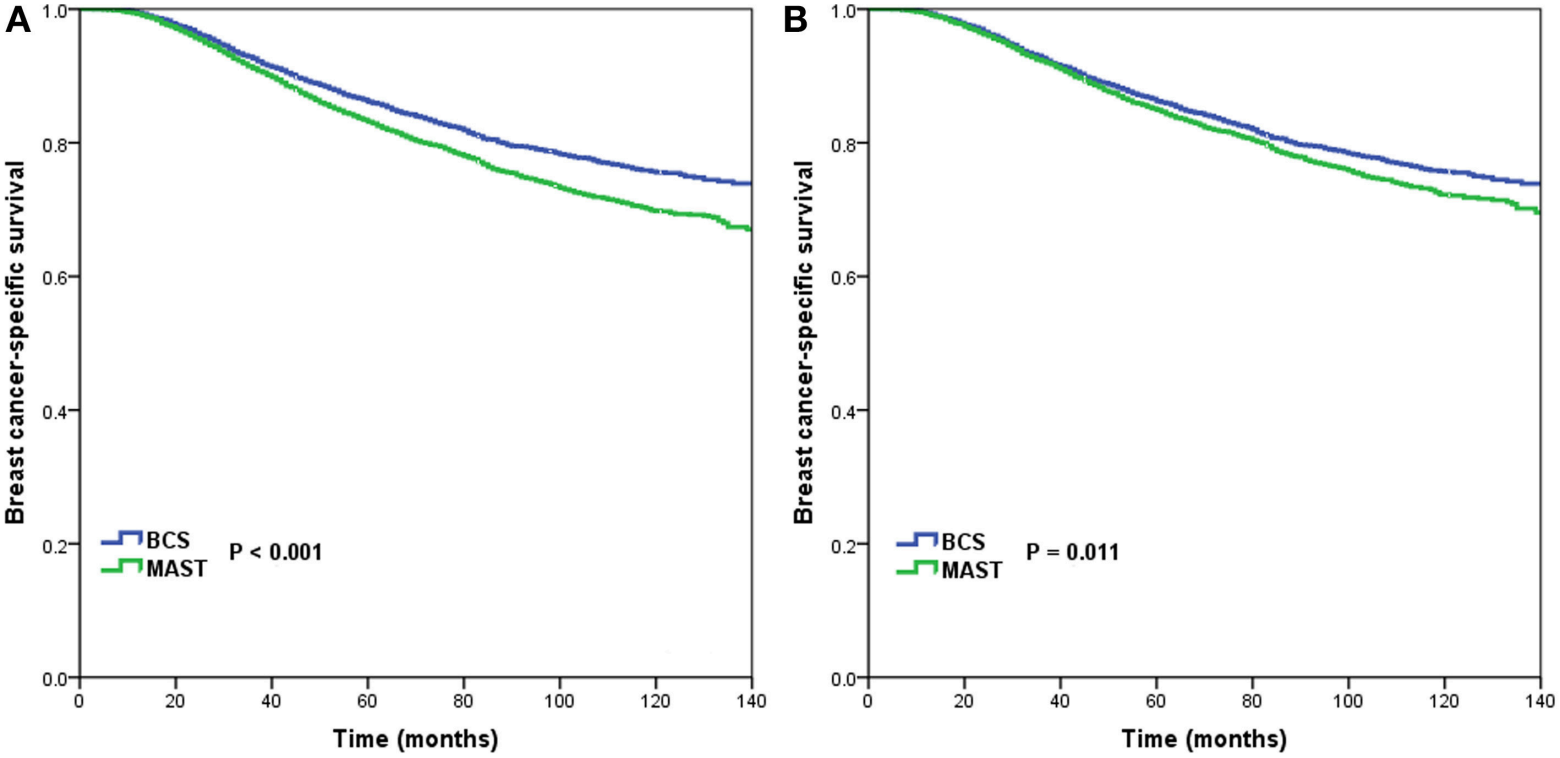
Breast cancer-specific survival after breast-conserving surgery and mastectomy before **(A)** and after **(B)** propensity score matching.

A total of 2,728 breast cancer-related deaths were observed during a median follow-up duration of 71 months (range, 3–143 months). The 5-year and 10-year BCSS was 84.2 and 72.0%, respectively. Kaplan-Meier analysis and the log-rank test showed significantly higher crude 5-year BCSS for BCS than for MAST (86.1 vs. 83.1%, *p* < 0.001) (Figure 3A).

### Multivariate Analysis Findings Before PSM

**Figure 4:**
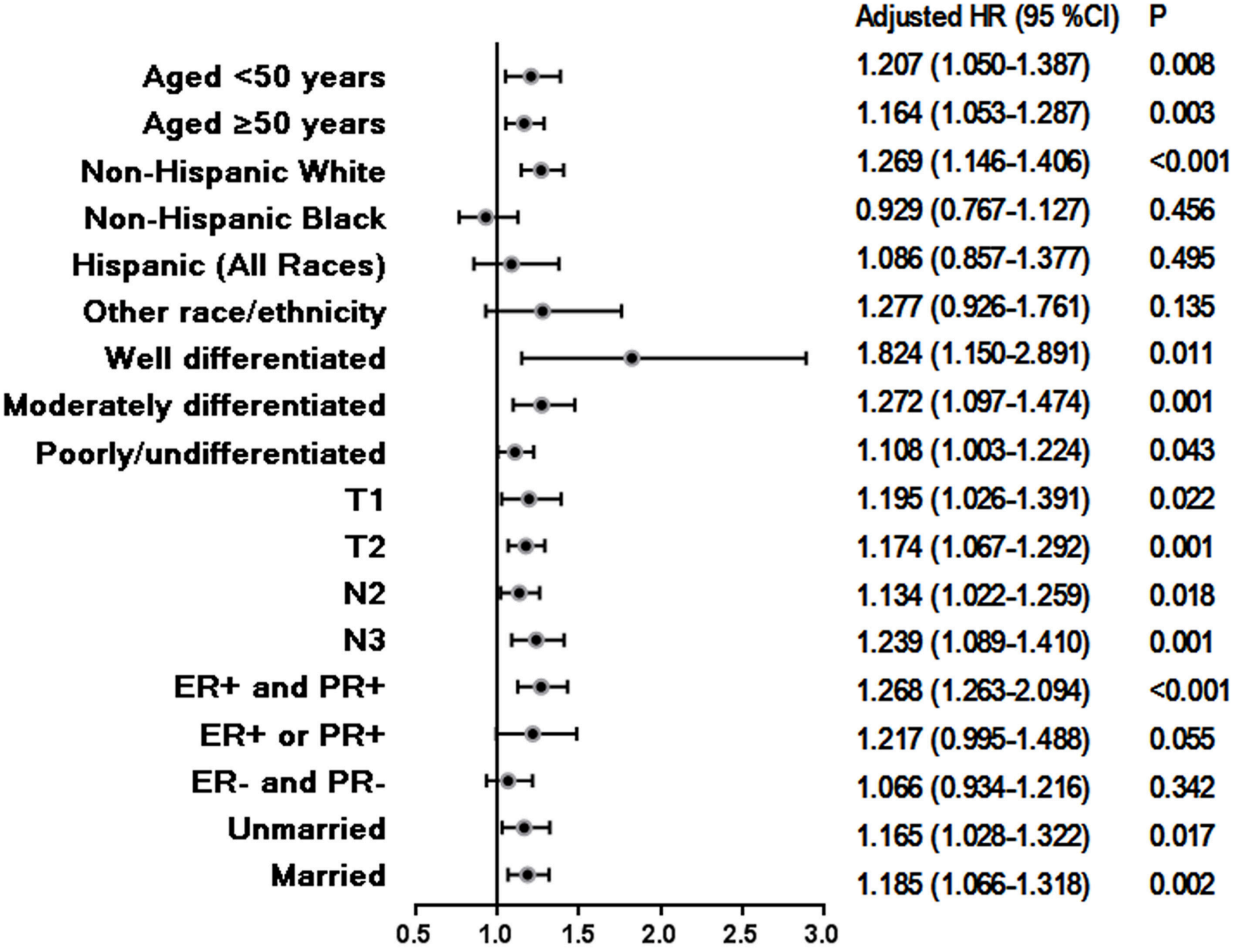
Hazard ratios for breast-conserving surgery compared to mastectomy with regard to breast cancer-specific survival in relation to predefined prognostic factors.

After the data were adjusted for age, race/ethnicity, grade, T stage, N stage, and HoR status as well as marital status, surgical procedure was found to be an independent prognostic factor associated with BCSS. Further, patients who received MAST had a worse BCSS than those treated with BCS (HR = 1.179, 95% CI = 1.087–1.278, *p* < 0.001). Multivariate analysis also showed that race/ethnicity, tumor grade, T stage, N stage, HoR status and marital status were independently associated with BCSS (Table 2). These results remained significant after stratification by age, tumor grade, T stage, N stage as well as marital status. After stratification for race/ethnicity, MAST showed worse BCSS than BCS in non-Hispanic white patients, but it showed similar BCSS to BCS in non-Hispanic black, Hispanic (all races), and other races/ethnicities. Moreover, for ER+ and PR+ tumors, MAST had a worse BCSS than BCS, but no significant difference was found between MAST and BCS in ER+ or PR+ as well as ER- and PR- patients (Figure 4).

**Table 2:** Multivariate analysis of prognostic factors for BCSS before and after PSM.

**Variables**	**Before PSM**			**After PSM**		
	**HR**	**95%CI**	***p***	**HR**	**95%CI**	***p***
**AGE (YEARS)**						
< 50	1			1		
≥50	1.041	0.961–1.127	0.325	1.029	0.929–1.139	0.586
**RACE/ETHNICITY**						
Non-Hispanic white	1			1		
Non-Hispanic black	1.282	1.151–1.428	< 0.001	1.354	1.189–1.543	< 0.001
Hispanic (all races)	1.006	0.892–1.133	0.928	1.072	0.923–1.246	0.363
Other	0.836	0.720–0.971	0.019	0.844	0.687–1.036	0.105
**GRADE**						
Well differentiated	1			1		
Moderately differentiated	1.471	1.199–1.803	< 0.001	1.537	1.160–2.036	0.003
Poorly differentiated/undifferentiated	1.910	1.560–2.339	< 0.001	2.073	1.566–2.743	< 0.001
**TUMOR STAGE**						
T1	1			1		
T2	1.372	1.256–1.498	< 0.001	1.368	1.233–1.517	< 0.001
**NODAL STATUS**						
N2	1			1		
N3	1.682	1.558–1.816	< 0.001	1.617	1.466–1.784	< 0.001
**HORMONE RECEPTOR STATUS**						
ER+ and PR+	1			1		
ER+ or PR+	1.512	1.358–1.583	< 0.001	1.490	1.295–1.714	< 0.001
ER- and PR-	1.932	1.765–2.114	< 0.001	1.833	1.636–2.053	< 0.001
**MARITAL STATUS**						
Unmarried	1			1		
Married	0.861	0.797–0.931	< 0.001	0.865	0.784–0.954	0.004
**SURGICAL PROCEDURE**						
BCS	1			1		
MAST	1.179	1.087–1.278	< 0.001	1.124	1.023–1.236	0.015

*BCS, breast-conserving surgery; CI, confidence interval; ER, estrogen receptor; HR, hazard ratios; MAST, mastectomy; PR, progesterone receptor; PSM, propensity score matching; T, tumor*.

### PSM Findings

Using PSM, 4,518 completely matched pairs were obtained. Kaplan-Meier analysis and the log-rank test showed a significantly higher crude BCSS for BCS than for MAST (75.7% vs. 72.2%, *p* = 0.011) (Figure 3B). After the data were adjusted for age, race/ethnicity, grade, T stage, N stage, HoR status as well as marital status, surgical procedure was found to be an independent prognostic factor related to BCSS. Further, patients who had undergone MAST had a worse BCSS than those who had undergone BCS (HR = 1.124, 95% CI = 1.023–1.236, *p* = 0.015) (Table 2).

## Discussion

In this study, we used a population-based analysis to assess the treatment outcome of BCS and MAST in patients with stage T1-2N2-3 breast cancer. Our results indicate that patients who underwent BCS had a better BCSS than those who underwent MAST before and after PSM was applied.

As expected, in the present study, most breast cancer patients with N2-3 disease were treated with MAST, similar to the findings of previous studies (2, 14, 17, 18). In addition, the rate at which MAST is performed gradually increased over the years—from 63.1% in 2004 to 79.8% in 2012; the increase can be mainly attributed to contralateral and bilateral mastectomy and breast reconstruction. Our results were similar to those of a study by the National Cancer Data Base (22). Although postoperative RT has a potential impact on breast reconstruction (23–25), in the present study, the number of patients who underwent reconstruction increased from 16.2% in 2004 to 28.2% in 2012. A prior SEER study also showed an increasing trend in breast reconstruction in patients who are suitable for postoperative RT (26). In China, although BCS and breast reconstructive surgery for N2-3 stage cancer have been increasing, only 5.4 and 1.9% of patients receive BCS and breast reconstruction, respectively (4). Fear of tumor recurrence may be an important factor that influences the preference for MAST, as it is perceived to reduce the need for repetitive surgery (27).

A population-based study from the Netherlands found better survival outcomes with BCS than with MAST in T1-2N2 patients diagnosed in 1999–2005, but such superior survival outcomes were not found in a 2006–2012 cohort of the same study (14). However, in their study, 16.2 and 20.8% of patients in the BCS and MAST group did not receive adjuvant systemic therapy, respectively, and 15% of the patients were not treated with postoperative RT (14). Another study from Canada found that the 5-year BCSS was 72.9 and 89.8% in patients with stage III disease who received MAST and BCS, respectively (2). However, nearly 50% of the patients did not receive adjuvant chemotherapy, and only one-third of the MAST patients were treated with adjuvant RT (2). Insufficient adjuvant treatment was also an observation in another Dutch study of the Netherlands Cancer Registry, which showed better overall survival for BCS than for MAST in patients with T2N2 disease, but not in patients with T1N2 disease (14). To ascertain the efficacy of BCS in patients with T1-2N2-3 breast cancer, our study assessed the non-inferiority of survival outcomes of BCS+chemotherapy+RT compared to MAST+chemotherapy+RT in a national cancer registry. The findings of our study also show that patients who received BCS had better BCSS than those who underwent MAST, but in contrast to all the previous studies, the patients included in our study received adjuvant treatment in accordance with the current clinical chemotherapy and radiotherapy practices. Our study therefore makes a valuable contribution to the current clinical practice of surgical treatment for N2-3 breast cancer patients.

Our subgroup analysis suggested that the BCSS was better with BCS than with MAST, regardless of age, grade, T stage, N stage as well as marital status. N3 stage disease is often considered a contraindication for BCS (16). However, we found that even in patients who have a high risk of locoregional and distant recurrence, BCSS is significantly better with BCS than with MAST. In addition, we found that ER+ or PR+ patients as well as ER- and PR- patients had poorer BCSS than ER+ and PR+ patients. Further, in ER+ or PR+ and ER- and PR- patients, BCSS was similar in the BCS and MAST groups. A prior study also showed that single HoR-positive breast cancer had similar outcomes to triple-negative breast cancer (28). These findings indicate that contemporary chemotherapy regimens may not maintain the survival advantage of BCS over MAST in these subgroups.

It is not entirely clear why BCS had a better outcome than MAST in the current cohort, but here are a few possible reasons. First, patients treated with BCS may have better self-image and sense of sexuality, which was related to better psycho-social wellbeing, better psychological health, and a higher level of satisfaction with life than those treated with MAST (29, 30). A prior study has indicated that a better quality of life was associated with better outcome after breast cancer treatment (31). This might explain why although the patient-reported cosmetic satisfaction is similar between BCS and breast reconstruction after MAST, the breast reconstruction cohort has a worse patient-reported outcome when postoperative RT is administered (23–25). Second, patients who receive BCS are more likely to be treated by experienced surgeons with academic affiliations (32), and the decision to perform BCS and associated surgical procedures may be run through a decision board group (33). Yet another reason could be that patients who receive BCS have better treatment compliance as a result of better health, and they may also have greater access to post-treatment medical surveillance. Finally, MAST is a more extensive and aggressive surgical method that may result in more adverse effects, including a more pronounced inflammatory response and tissue damage, which could negatively affect the immune system and promote residual tumor cell growth (34, 35).

The primary strengths of our study are its population-based characteristics including the large cohort of patients and stratification for demographic and clinicopathological characteristics. However, we need to acknowledge several limitations of this study too. First, our study was a retrospective study, which means that several confounding factors and potential selection biases cannot be ruled out, even though we used propensity score analysis. However, since there are no randomized controlled trials that compare the outcomes of BCT and MAST in patients with high-risk breast cancer, our study reported the outcome from real-world data. We believe that the findings are important in light of daily treatment practices for breast cancer surgery. Second, the SEER dataset does not include detailed information regarding the chemotherapy regimen, endocrine therapy, anti-HER2 targeted therapies, or sequential surgery and chemotherapy data, which may limit the generalizability of our findings. Third, the details of RT regarding RT technique, target volume, RT dose, and tumor bed boost between the two groups were also lacking in the SEER database. Finally, the patterns of disease recurrence and treatment history after tumor recurrence were not captured in the SEER program.

## Conclusion

In conclusion, the findings of our study indicate that patients with T1-2N2-3 invasive breast cancer who undergo BCS have a noninferior outcome than those who receive MAST. Given that MAST is a more invasive surgical procedure that has more sequelae than BCS, we believe that BCS may be an optimal treatment option when both treatment options are feasible and appropriate.

## Ethics Statement

The study was exempt from the approval processes of the Institutional Review Boards because the SEER database patient information is de-identified.

## Author Contributions

S-GW aided in data collection. JW, J-PD, YD, J-YS, and W-WZ are authors who participated in manuscript drafting, table/figure creation, and manuscript revision. S-GW and Z-YH are the corresponding authors who initially developed the concept and drafted and revised the manuscript. All authors read and approved the final manuscript.

### Conflict of Interest Statement

The authors declare that the research was conducted in the absence of any commercial or financial relationships that could be construed as a potential conflict of interest.
